# Organ-specific transcriptome profiling of metabolic and pigment biosynthesis pathways in the floral ornamental progenitor species *Anthurium amnicola* Dressler

**DOI:** 10.1038/s41598-017-00808-2

**Published:** 2017-05-04

**Authors:** Jon Y. Suzuki, Teresita D. Amore, Bernarda Calla, Nathan A. Palmer, Erin D. Scully, Scott E. Sattler, Gautam Sarath, Joanne S. Lichty, Roxana Y. Myers, Lisa M. Keith, Tracie K. Matsumoto, Scott M. Geib

**Affiliations:** 1 USDA Agricultural Research Service, Daniel K. Inouye U.S. Pacific Basin Agricultural Research Center, Hilo, HI 96720 USA; 2 Department of Tropical Plant & Soil Sciences, College of Tropical Agriculture and Human Resources, University of Hawaii at Manoa, 3190 Maile Way Rm. 102, Honolulu, HI 96822 USA; 3 USDA Agricultural Research Service, Wheat, Sorghum, and Forage Research Unit, Lincoln, NE 68583 USA; 4 USDA Agricultural Research Service, Stored Product Insect and Engineering Research Unit, Manhattan, KS 66502 USA; 5 Department of Agronomy and Horticulture, University of Nebraska-Lincoln, Lincoln, NE 68583 USA; 6 Department of Entomology, University of Illinois at Urbana-Champaign, Urbana, IL 61801 USA

## Abstract

*Anthurium amnicola* Dressler possesses a number of desirable and novel ornamental traits such as a purple-colored upright spathe, profuse flowering, and floral scent, some of which have been introgressed into modern *Anthurium* cultivars. As a first step in identifying genes associated with these traits, the transcriptome from root, leaf, spathe, and spadix from an accession of *A*. *amnicola* was assembled, resulting in 28,019 putative transcripts representing 19,458 unigenes. Genes involved in pigmentation, including those for the metabolism of chlorophyll and the biosynthesis of carotenoids, phenylpropanoids, and flavonoids were identified. The expression levels of one MYB transcription factor was highly correlated with *naringenin 3*-*dioxygenase* (*F3H*) and *dihydroflavonol*-*4*-*reductase* (*DFR*) in leaves, whereas a bHLH transcription factor was highly correlated with *flavonoid 3*′-*monooxygenase* (*F3*′*H*) and a *DFR* in spathes, suggesting that these two transcription factors might regulate flavonoid and anthocyanin synthesis in *A*. *amnicola*. Gene sequence and expression data from four major organs of *A*. *amnicola* provide novel basal information for understanding the genetic bases of ornamental traits and the determinants and evolution of form and function in the Araceae.

## Introduction

*Anthurium* is a major cut flower and potted plant with a long and important history of breeding, selection, and cultivation in Hawaii. Today, the largest production comes from the Netherlands, with significant production and research in Hawaii and other tropical and subtropical areas. A popular attraction is its colorful spathe, a modified leaf that is associated with the spadix, an organ often also found in varied colors housing numerous bisexual, protogynous flowers. The major spathe color classes red, pink, orange, coral, and purple are from variations in anthocyanin type and levels and white coloration represents loss of pigment. Further, green coloration is thought to occur from variations in chlorophyll production, while brown coloration is likely derived from variations in anthocyanin and chlorophyll content^1–5^.

The genus *Anthurium* consists of as many as 1,500 species found between Southern Mexico and Northern Argentina and is the largest genus in the Araceae family^6^. A limited sampling revealed that *Anthurium* species possess medium-sized genomes ranging from between 2 to 10 Gbp per haploid genome^7^. A majority of the modern *Anthurium* cultivars in the market today descend from historical or recent hybrids between species^8^ particularly with *A*. *andraeanum*, which is characterized by the commercially popular heart-shaped spathe. Spathe and spadix color and their genetic inheritance were some of the first traits to be studied^9–11^. The orange and red colors may have arisen from the activation of two different branches of the anthocyanin biosynthetic pathway, which are potentially controlled by two different transcription factors. Plants harboring recessive alleles for two loci produced white spathes, although the actual genes coding for these putative regulatory protein factors were not identified^10, 11^. The genetic mechanisms linked to spathe and spadix color continue to be priority research areas in addition to other ornamental characteristics related to spathe and spadix form^8, 12^.

The relatively long generation times (2–3 years) for most *Anthurium*, limited information on cultivar pedigrees, interspecific sterility, and the paucity of mapping populations^13^ remain major constraints toward cultivar development and genetic analyses. The recent publication of several *Anthurium* transcriptomes of whole plants, whole floral tissues, leaves, or spathes, and the genomes and/or transcriptomes from a number of aroids has enabled a first glimpse at the gene repertoire of this plant family^14–19^, supplanting gene expression studies of individual pathways in *Anthurium* ^11, 19–22^ and supporting further development of molecular tools for ornamental and horticultural improvement (see ref. 23 for review).

**Figure 1:**
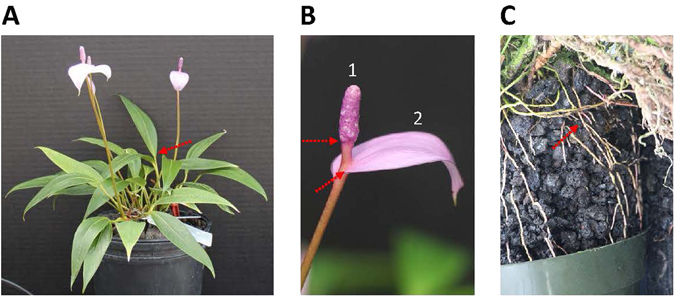
*Anthurium amnicola* morphology and sampling. (**A**) Whole plant, (**B**) 1. spadix, 2. (recurved) spathe, and (**C**) subterranean roots. Red dashed arrows indicate dissection points for sampling leaves, spadix and spathe, and roots from 1A, 1B and 1C, respectively.

*Anthurium amnicola* Dressler possesses several horticulturally important traits, including a purple spathe and spadix and floral scent, although the genes responsible for these traits have yet to be identified. However, the novel purple spathe and spadix is associated with the accumulation of the anthocyanin peonidin^24^, which is characteristic of this species, and has been introgressed into modern hybrids. In the current study, transcriptome analysis from four major organs of *A*. *amnicola* (Fig. 1) was performed with the aim of identifying genes of ornamental and horticultural importance and their characteristic expression patterns among the different organs in their original, species context.

## Results

### Sequencing and quality filtering

In total, approximately 234.8 million 151 × 151 read pairs were obtained from Illumina HiSeq 2500 sequencing, totaling over 70.9 Gb of data (Supplementary Table S1). *A*. *amnicola* organ samples from which sequence data were derived were all harvested within the same period and day from clonal plants grown under shadehouse conditions typical for *Anthurium* (see Methods). *A*. *amnicola* spathe and spadix samples were derived from floral stems that emerge at a rate of approximately 9.5 per year per growing point and represented a narrow developmental window (see Methods). Reads were generally evenly distributed between all of the sample libraries sequenced, ranging from 17.26 to 23.65 million reads per sample. After trimming low quality bases and removing low quality reads, approximately 95% of the read pairs remained and were used for assembly and mapping, representing 222.5 million read pairs and 67.2 Gb of data (Supplementary Table S1). *In silico* normalization using the Trinity normalization tool greatly reduced the computational requirements for assembly and removed complex de Bruijn graphs created by kmers derived from low quality or overly abundant sequences without negatively impacting k-mer diversity. From the 234,798,577 raw read pairs, normalization reduced the read pair abundance to 12,198,122 (~5.2% of total), which were used as input into Trinity.

### *De novo* transcriptome assembly and transcript filtering

The raw *A*. *amnicola* transcriptome assembly yielded 499,693 putative transcripts across 412,974 unigenes with an N50 transcript size of 1,015 base pairs and a transcript sum of 345.32 Mb (Supplementary Table S2). Filtering based on read abundance, component isoform percentage, and presence of an open reading frame to retain high quality protein coding transcript isoforms reduced the assembly to 27,959 transcripts across 19,420 unigene models. While this does not represent the entire gene set for this species, these results are reasonable, considering only four organ types were sampled from a limited number of developmental stages. Complete ORFs for 14,108 (50.5%) of the retained transcripts, representing 9,781 (50.3%) of the final unigene set, were predicted. The N50 transcript size of this filtered assembly was 2,247 bp with a transcript sum of 52.5 Mb using all transcripts and 35.8 Mb when only the longest isoform for each unigene was taken into consideration (Supplementary Table S2).

**Figure 2:**
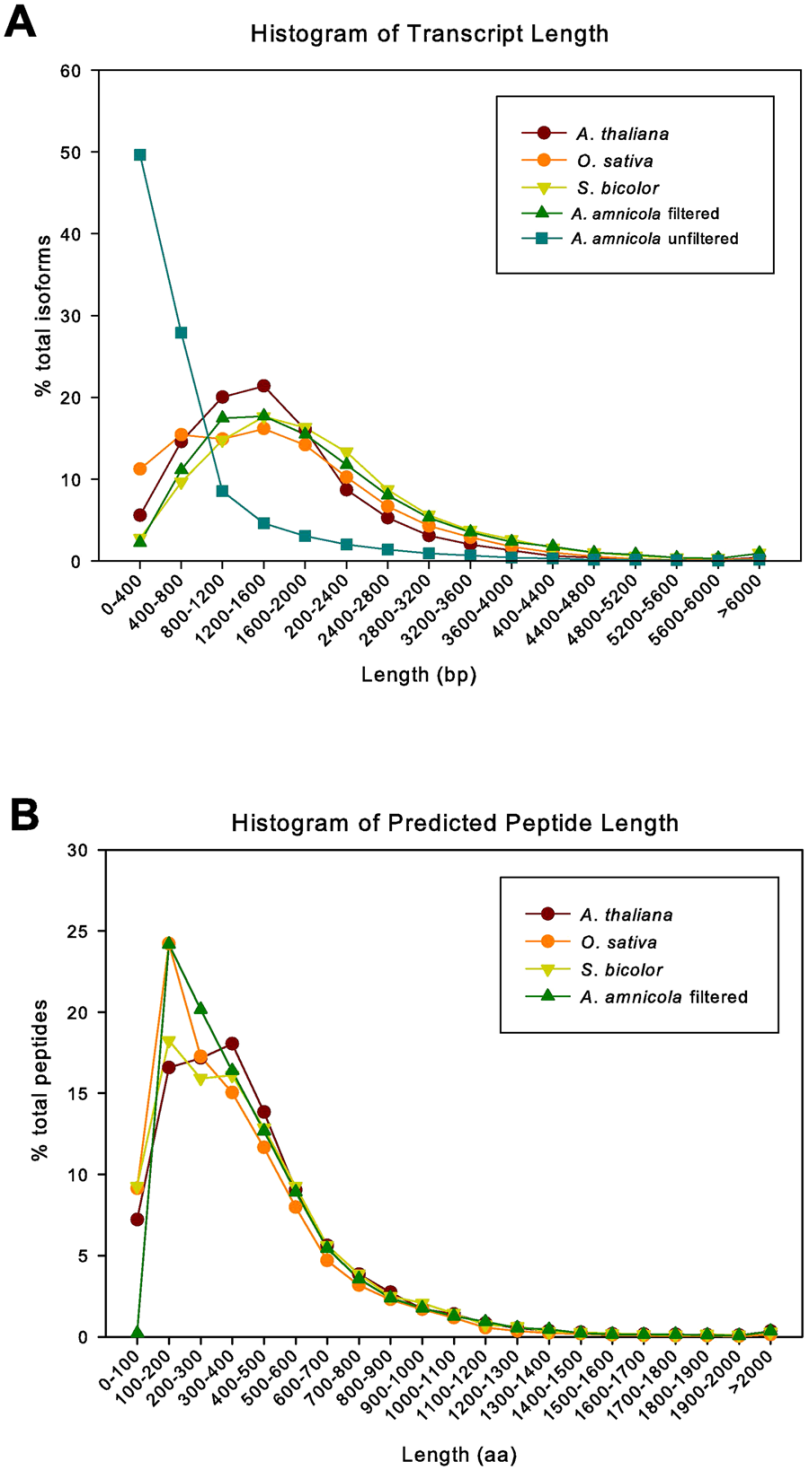
*A*. *amnicola* transcriptome assembly transcript and peptide length compared to current gene sets from several established plant genomes. Distribution of (**A**) transcript length and (**B**) predicted protein length of the *A*. *amnicola* transcriptome compared to published transcript and protein sets from *A*. *thaliana*, *O*. *sativa*, and *S*. *bicolor* (obtained from Phytozome version 10.3; with genome releases Athaliana_167_TAIR10, Osativa_204_v7.0 and, Sbicolor_255_v2.1).

The distribution of transcript lengths of the filtered *A*. *amnicola* transcriptome assembly more closely resembled the distribution of transcript lengths in the *Arabidopsis thaliana*, *Oryza sativa*, and *Sorghum bicolor* genomes compared to distribution of the unfiltered assembly (Fig. 2A). In addition, the predicted peptide lengths derived from the filtered assembly also resembled the predicted peptide length distribution in the same three plant genomes (Fig. 2B).

### Functional annotation

A large proportion of the transcripts retained after filtering could be functionally annotated based on BLASTp^25–27^ matches of their predicted open reading frames to proteins in the UniProt SwissProt/KB database as well as peptide searches through InterProScan. 26,384 of the total transcripts (94.3%) corresponding to 18,688 (96.2%) of the total predicted unigenes had at least one BLASTp match to a SwissProt protein (Supplementary Table S2). 21,863 (78.2%) of the transcripts corresponding to 15,303 (78.8%) of the unigene models contained an annotated Pfam domain, representing 5,528 unique Pfam accessions. In addition, 17,294 transcripts corresponding to 12,100 unigenes were assigned at least one gene ontology (GO) term representing 2,210 distinct GO annotations, and KEGG (Kyoto Encyclopedia of Genes and Genomes)^28, 29^ orthology (KO) terms and metabolic pathways were predicted for 5,136 transcripts (18.3% of the total) corresponding to 4,335 unigenes (22.3% of the total).

To assess the completeness of the transcriptome assembly, the number of *A*. *amnicola* transcripts encoding KEGG pathway enzymes was determined (Supplementary Table S3A) and compared to numbers of enzymes detected in the genomes of several well-studied plant species, including *Sorghum bicolor* (sorghum), *Oryza sativa* (rice), and *Arabidopsis thaliana* (Arabidopsis). To avoid biases caused by species-specific duplications of certain pathway enzymes, each KO term was counted only once in the *A*. *amnicola* transcriptome and the other three plant genomes (Supplementary Table S3B). The resulting number of unique KO terms from the *A*. *amnicola*, sorghum, rice, and Arabidopsis were calculated to be 2,293, 2,490, 2,498, and 2,691, respectively. The data indicate that several conserved metabolic pathways were well represented in the assembly, including glycolysis (30 unique KO terms in *A*. *amnicola* compared to 26, 27, and 27 KO unique terms in the sorghum, Arabidopsis, and rice genomes respectively), the TCA cycle (16 unique KO terms in sorghum and *A*. *amnicola* compared to 19 KO unique terms in rice and Arabidopsis), and carbon fixation (22, 19, 23, and 21 unique KO terms in *A*. *amnicola*, sorghum, rice, and Arabidopsis, respectively). Other pathways well-represented in the *A*. *amnicola* dataset included starch and sucrose metabolism, biosynthesis of amino acids, purine metabolism, and pyrimidine metabolism. KO assignments for conserved metabolic pathways could be identified suggesting that the *A*. *amnicola* transcriptome contains a comprehensive representation of a majority of the important metabolic pathway genes.

### Major organ type gene expression patterns and sample validation

**Figure 3:**
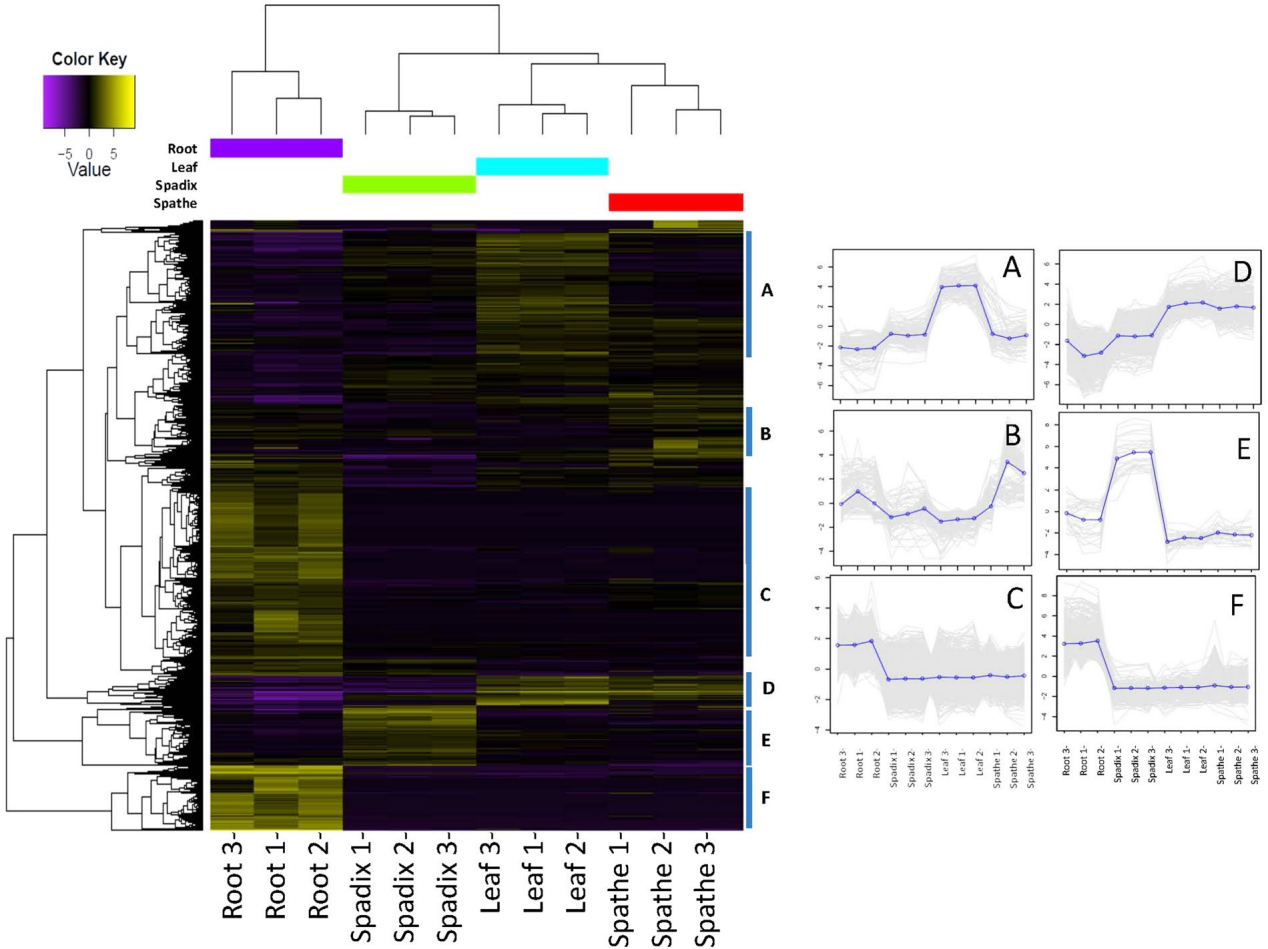
Expression profiles of *A*. *amnicola* unigenes across four major organs. Two dimensional clustering of organ types by expression patterns of the 10,440 differentially expressed unigenes. Cluster expression profiles are presented as inset figures (**A**–**F**). Vertical axis of inset figures is log_2_ (median centered FPKM values).

Differential expression analyses aided by hierarchical clustering and annotation provided an overall visualization of the expression profiles across organs. For a general overview of the differential expression results calculated with EdgeR, a hierarchical cluster analysis was performed using expression levels from the 10,440 unigenes (FDR corrected p-values < 0.05 and log_2_ fold change >1.5) that were identified as differentially expressed in at least one of the four organ types (Fig. 3, Supplementary Table S4). For these analyses, the three spathe and three spadix samples were each considered as biological replicates of their respective organ types, despite being collected at different developmental stages. While we anticipate variability between these replicate samples, the hierarchical clustering results provided support for replicate sample verity by correct clustering by organ type. This result, thus also suggested that general descriptions on gene expression and pattern differences between organs regardless of developmental differences among replicate samples could be made in subsequent analyses of the data. By partitioning the clustering of gene expression profiles, groups of genes expressed in similar patterns were identified that were descriptive of organ specific expression patterns (Fig. 3, inset A–F). Unigenes found in cluster A, 41% of which were annotated as chloroplastic origin, were expressed higher in leaves than any other organ. Two clusters (Fig. 3, inset C and F) contained unigenes expressed highly in roots. Clustering analysis also identified a group of unigenes with higher expression values in spathes and leaves compared to spadices and roots (Fig. 3, Inset D); about 12% of these unigenes were annotated as being of chloroplastic origin, and most had slightly higher expression levels in leaves compared to spathes. A small cluster of unigenes with highest expression in the spadix was observed (Fig. 3, inset E) as well as a cluster of genes that exhibited highest expression in the spathe (Fig. 3, inset B).

### Identification of transcripts expressed in an organ-biased manner

**Figure 4:**
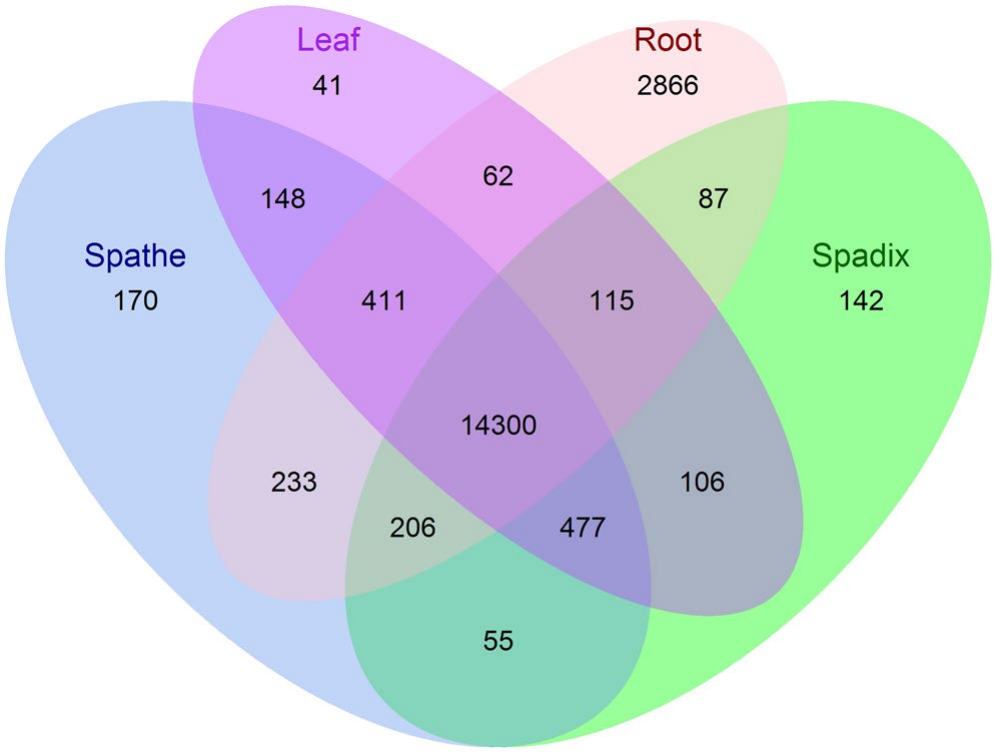
Venn diagram of *A*. *amnicola* expressed unigene number by organ. Unigenes expressed in roots, spathe, spadix, and leaves with maximum FPKM values ≥1 in one or more of the three biological replicates were defined as being expressed. Unigenes with maximum FPKM values = 0 for each replicate of a particular organ were defined as not being expressed in that organ.

To conservatively identify unigenes that were likely expressed in an organ-biased manner, the maximum FPKM (fragments per feature kilobase per million reads mapped) expression values of all of the biological replicates analyzed for each organ type were utilized (Fig. 4). If the FPKM value was ≥1 in a given organ, it was classified as being expressed; however, if this value was equal to 0, it was classified as not being expressed. Using these criteria, the majority of unigenes (14,300) were expressed in all organs while few unigenes were exclusively expressed in the spathe (170; 0.88% of the total unigenes), spadix (142; 0.73%), and leaf (41; 0.21%). The root had the largest number of uniquely expressed unigenes with 2,866 (14.8%).

Large percentages of the unigenes detected exclusively in the root were involved in transcriptional and translational machinery (i.e., ribosomal proteins, transcription factors, RNA polymerase, elongation factors, and translation initiation factors), signal transduction, and nutrient and mineral assimilation (i.e., ABC transporters, ammonium transporters, aquaporins, copper transporters, magnesium transporters, phosphate transporters, and sugar transporters). In addition, 45 transcripts predicted to encode cytochrome P450 enzymes were expressed exclusively in the root.

The spathe expressed a slightly larger number of unigenes in an organ-biased manner compared to the spadix; three are plant-specific transcription factors, including two transcripts annotated as NAC transcription factors and one annotated as a TIFY transcription factor. A single unigene encoding chalcone synthase, the first committed step in flavonoid biosynthesis, was also expressed specifically in the spathe. Other unigenes expressed specifically in the spathe could be linked to stress response, including heat shock proteins, stress-associated endoplasmic reticulum proteins, and peptidoglycan binding proteins.

The spadix exclusively expressed 142 unigenes, including 20 transcription factors (10 MADS-box transcription factors, five MYC transcription factors, and five MYB transcription factors), multiple homeotic and developmental unigenes (encoding five homeobox proteins and five floral homeotic proteins), and several unigenes involved in the biosynthesis of lipids and polyaromatic compounds. For example, seven unigenes annotated as omega-hydroxypalmitate-O-feruloyl transferases, five unigenes annotated as GDSL esterase/lipases, and five unigenes annotated as fatty acyl-CoA reductases were exclusively expressed in the spadix. These unigenes are probably involved in the biosynthesis of cutin glycerolipid polymers^30^. Finally, 55 unigenes were expressed in both the spathe and spadix, including five cinnamoyl-CoA reductases (CCRs) associated with phenylpropanoid biosynthesis, four chloroplastic polyphenol oxidases, and several regulatory unigenes (five G-type lectin S-receptor-like serine/threonine-protein kinases, two receptor-like serine/threonine protein kinases, one MYB transcription factor, and one zinc finger protein). In addition, two unigenes annotated as momilactone-A synthase and six unigenes annotated as jacalin-related lectin proteins were exclusively expressed in these two tissues. Momilactone-A synthases and similar genes have been annotated among sequences from numerous plants, and their putative product momilactone, a defensive compound produced in response to elicitor signals, has only been recognized in a few plants to date^31^. Jacalin-related lectin proteins perform unidentified roles in plant immunity, defense, and response to biotic and abiotic stresses^32^.

### Expression levels of unigenes assigned to core metabolic pathways across organ type

**Figure 5:**
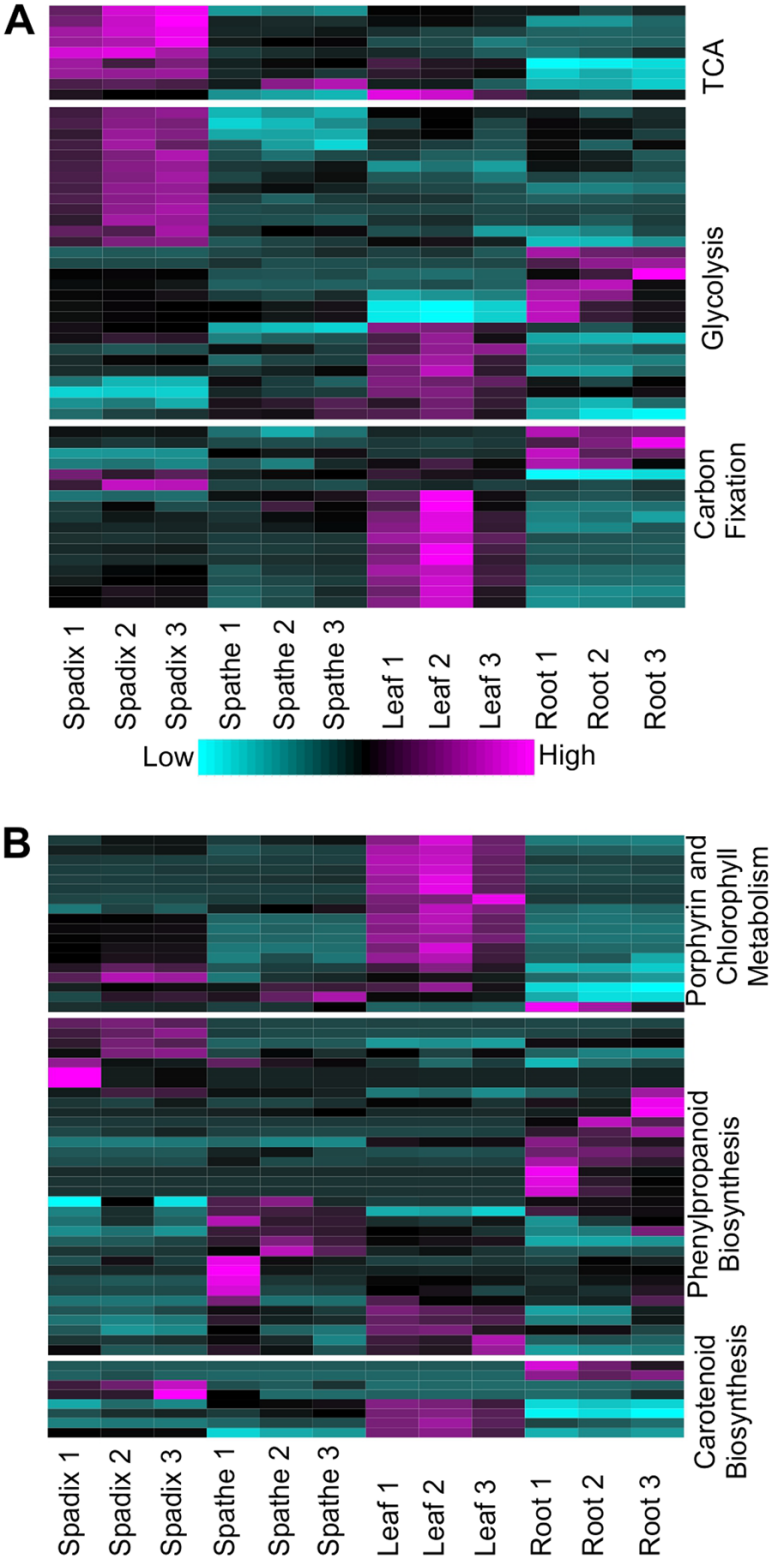
Expression patterns of *A*. *amnicola* unigenes assigned to metabolic and pigment biosynthetic pathways in spadix, spathe, leaf, and root. (**A**) Differentially expressed unigenes found in the TCA cycle, glycolysis, and carbon fixation pathways. (**B**) Differentially expressed unigenes assigned to porphyrin and chlorophyll metabolism, phenylpropanoid biosynthesis, and carotenoid biosynthesis pathways.

A majority of differentially expressed unigenes assigned to the tricarboxylic acid (TCA) cycle and glycolysis pathways had the highest expression levels in the spadix with smaller numbers of unigenes in these pathways expressed in the leaf and root as shown by heatmap analysis of the replicate *A*. *amnicola* organ samples (Fig. 5A).

Nearly every differentially expressed unigene assigned to the TCA cycle was more highly expressed in the spadix compared to the roots, leaves, and spathes; however, one TCA cycle unigene encoding succinate dehydrogenase was highly expressed in the spathe while one unigene encoding dihydrolipoamide dehydrogenase was most highly expressed in leaves. Likewise, over 15 differentially expressed unigenes assigned to the glycolysis pathway were most highly expressed in the spadix while seven unigenes each were most highly expressed in leaves and roots.

More than 50% of the differentially expressed unigenes classified as carbon fixation genes were most highly expressed in leaf tissue including a *malate dehydrogenase*, *fructose*-*1*,*6*-*bisphosphatase I*, two *glyceraldehyde 3*-*phosphate dehydrogenases*, *triosephosphate isomerase*, and *ribulose*-*phosphate 3*-*epimerase*. In addition, four unigenes classified as carbon fixation genes, including *pyruvate orthophosphate dikinase* and *phosphoenolpyruvate carboxylase*, were most highly expressed in the roots while two, *malate dehydrogenase* and *fructose-bisphosphate aldolase*, were most highly expressed in the spadix. The two unigenes more highly expressed in spadix are also known components of other metabolic pathways including glycolysis and the TCA cycle and are distinct from carbon fixation unigenes most highly expressed in the leaves.

Higher expression levels of glycolysis and TCA pathway unigenes in the spadix suggest that floral tissue actively undergoing anthesis and nectar and scent production might be significant energy sinks, relative to other parts of the plant.

### Expression patterns of unigenes involved in the metabolism of chlorophyll and the synthesis of phenylpropanoids and carotenoids across organ type

Expression levels of differentially expressed unigenes assigned to the porphyrin and chlorophyll metabolism pathway and the phenylpropanoid and carotenoid biosynthetic pathways in the four *A*. *amnicola* organs were also compared by heatmap analysis (Fig. 5B). Two unigenes assigned to the porphyrin and chlorophyll metabolic pathway were most highly expressed in the spadix, including *uroporphyrinogen*-*III synthase* and *pheophorbide a oxygenase*. In addition, *red chlorophyll catabolite reductase* and *chlorophyllase* were highly expressed in all aerial tissues.

Seven unigenes associated with phenylpropanoid biosynthesis were highly expressed in the spadix compared to other organs (Fig. 5B). One of these unigenes was annotated as *4*-*coumarate*-*CoA ligase* (*4CL*), which catalyzes the formation of 4-coumaroyl CoA, a substrate used for the synthesis of monolignols and flavonoids. While 4-coumaric acid is typically the preferred substrate for 4CL involved in lignin biosynthesis, 4CLs can also accept other compounds as substrates, such as caffeic acid and ferulic acid, albeit at a lower efficiency^33^. Unigenes coding for cinnamyl alcohol dehydrogenase (CAD; one copy) and shikimate O-hydroxycinnamoyltransferase (HST; one copy) were also strongly expressed in spadix compared to the other three organs. Although specific 4CL, HST, and CAD enzymes catalyze steps in the monolignol biosynthesis branch of the phenylpropanoid biosynthesis pathway, nonlignifying, 4CLs, HSTs, and CADs can utilize a variety of substrates and have been implicated in pathways leading to production of volatile aromatics^34, 35^. Finally, a unigene annotated as *coumaroylquinate* (*coumaroylshikimate*) *3*′-*monooxygenase* was also significantly expressed in the spadix, which can catalyze the formation of chlorogenic acid or 5-O-caffeoyl shikimic acid. Both chlorogenic acid and 5-O-caffeoyl shikimic acid have roles in defense against biotic stresses. In the spathe, the expression levels of seven peroxidases, three β-glucosidases, and one 4CL associated with phenylpropanoid metabolism were strongly expressed.

Four unigenes coding for enzymes involved in carotenoid biosynthesis, including *lycopene epsilon*-*cyclase*, *phytoene synthase*, *violaxanthin de*-*epoxidase*, and *carotene epsilon*-*monooxygenase* were more strongly expressed in leaves compared to the other three organs. Lycopene epsilon-cyclase catalyzes the formation of provitamin A carotenoids, which are used for the synthesis of cyclic xanthophylls. Two unigenes assigned to this pathway were also more highly expressed in the spadix, including *9*-*cis*-*epoxycarotenoid dioxygenase* and *abscisic acid 8*′-*hydroxylase*, which are both involved in the metabolism of the plant hormone abscisic acid.

### Expression patterns of flavonoid biosynthesis unigenes in different organs

**Figure 6:**
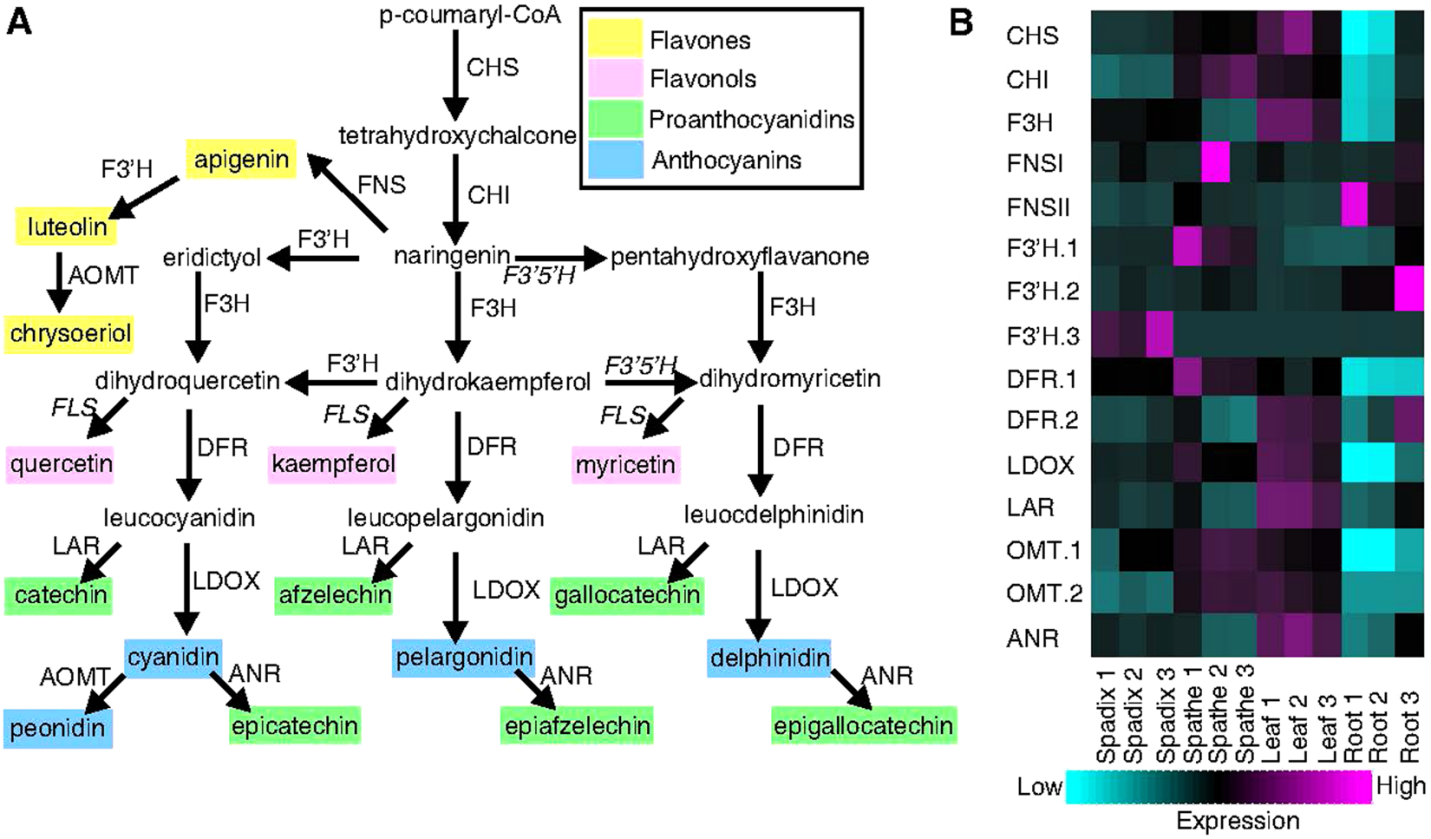
Flavonoid biosynthesis pathway and *A*. *amnicola* flavonoid unigene expression across organ type. (**A**) An overview of the flavonoid biosynthesis pathway enzymes and metabolites. Homologs for F3′5′H and FLS (in italics) could not be confidently identified in the transcriptome assembly. Abbreviations are as follows: CHS (chalcone synthase), CHI (chalcone isomerase), F3H (naringenin 3-dioxygenase), FNS (flavone synthase), DFR (dihydroflavonol 4-reductase), LDOX (leucoanthocyanidin dioxygenase), F3′H (flavonoid 3′-monooxygenase), F3′5′H (flavonoid 3′,5′-hydroxylase), FLS (flavonol synthase), LAR (leucoanthocyanidin reductase), AOMT (anthocyanidin O-methyl transferase), and ANR (anthocyanidin reductase). (**B**) A heatmap of the expression profiles of flavonoid biosynthesis pathway genes identified in the *Anthurium amnicola* transcriptome. Magenta is high expression, cyan is low expression. Unigene IDs are shown in Supplementary Table S5.

Metabolic pathways associated with plant coloration in *Anthurium* are of particular commercial and research interest. The flavonoid biosynthesis pathway is the primary route for producing pigments leading to colors ranging from light yellow to blue/purple in different plants^36–38^. Unigenes involved in flavonoid biosynthesis in the *A*. *amnicola* transcriptome assembly were identified using BLASTp with previously identified *Oryza sativa* genes annotated in KEGG and additional literature^39, 40^ (Supplementary Table S5). The enzymatic steps leading to the production of flavones, flavonols, proanthocyanidins, and anthocyanins are outlined in Fig. 6A. Putative unigenes for nearly all enzymes were identified in the *A*. *amnicola* transcriptome (Supplementary Table S5) the only exceptions being the homologs of rice *flavonol synthase* (*FLS*), a dioxygenase, and *flavonoid 3*′,*5*′-*hydroxylase* (*F3*′*5*′*H*), a cytochrome P450 enzyme. The potential lack of a *flavonol synthase* unigene was not expected, although to our knowledge this has not been reported from *Anthurium*. However, the genes(s) encoding F3′5′H are absent from many plants^41, 42^, and the associated 3′,5′ hydroxylated flavonoid compounds, such as delphinidin, have not been detected in numerous members of the *Araceae* family^43^ including *A*. *amnicola* ^3, 5^, suggesting that *F3*′*5*′*H* may not be expressed in this species. Plant genomes do contain a large number of dioxygenases and P450 unigenes, and the *A*. *amnicola* transcriptome assembly contains 22 dioxygenases (PF03171) and 154 P450s (PF00067). Thus, it is possible that a more distantly related gene could be coding for the *A*. *amnicola* FLS and F3′5′H.

The normalized expression profiles of all the putative flavonoid biosynthesis unigenes found in the *A*. *amnicola* transcriptome are shown in Fig. 6B. With the exception of *flavone synthase I* (*FNSI*), all of the identified unigenes were found to be differentially expressed in the data set. In general, most of the flavonoid biosynthesis unigenes, including unigenes specifically assigned to the anthocyanin biosynthesis pathway, had higher expression in leaf tissue (*CHS*, *F3H*, *DFR*.*2*, *LDOX*, *LAR*, and *ANR*) or in spathe tissue (*CHI*, *FNSI*, *F3*′*H*.*1*, *DFR*.*1*, *OMT*.*1*, and *OMT*.*2*) (Fig. 6B). Expression of the flavonoid biosynthesis genes in spathes and leaves is consistent with detection of various related flavonoids in spathes of *A*. *amnicola*, including anthocyanins, flavones, and the proanthocyanin epicatechin, the latter initially characterized from leaves of an *Anthurium* cultivar^5^. *FNSII* and *F3*′*H*.*2* had highest expression in root tissue while *F3*′*H*.*3* had maximal expression in spadix tissue (Fig. 6B). Interestingly, sample specific expression was observed for nearly all flavonoid pathway unigenes in the Root-3 sample compared to the Root-1 and Root-2 samples (Fig. 6B) and may be linked to the pigment noted for this specific root sample (Supplementary Table S1). Although we detected a number of flavonoid biosynthetic unigenes based on homology to flavonoid genes from rice, as mentioned above, metabolite analysis of *A*. *amnicola* spathes has shown a number of known stably accumulating flavonoids as well as a number of unknown intermediates^5^, and thus unigene assignment to specific flavonoid steps particularly for the genes involved in noncore enzymatic activities such as O-methyl transferase (OMT) are difficult to determine without direct enzymatic or genetic data.

### qPCR validation

One gene from each of the seven metabolic pathways, TCA, glycolysis, carbon fixation, porphyrin metabolism, phenylpropanoid biosynthesis, carotenoid biosynthesis and flavonoid biosynthesis (Supplementary Table S6), were analyzed by reverse transcription qPCR on RNA from the same samples used for RNA-Seq. Results for six genes encoding ATP citrate (pro-S)-lyase (TCA), dihydrolipoamide acetyltransferase (TCA/glycolysis), fructose-bisphosphate aldolase (carbon fixation/glycolysis), uroporphyrinogen-III synthase (porphyrin metabolism), 9-cis-epoxycarotenoid dioxygenase (carotenoid biosynthesis) and chalcone isomerase (flavonoid biosynthesis) were highly correlated with the RNA-Seq analysis (R^2^ ≥ 0.50) (Supplementary Fig. S1). The exception was *HST*, the phenylpropanoid biosynthesis gene encoding shikimate O-hydroxycinnamoyltransferase, the qPCR results of which did not significantly correlate with RNA-Seq data. These results suggest that qPCR analyses were for the most part confirmatory of the general expression patterns represented by RNA-seq data for this sampling of target genes.

### Network analysis

**Figure 7:**
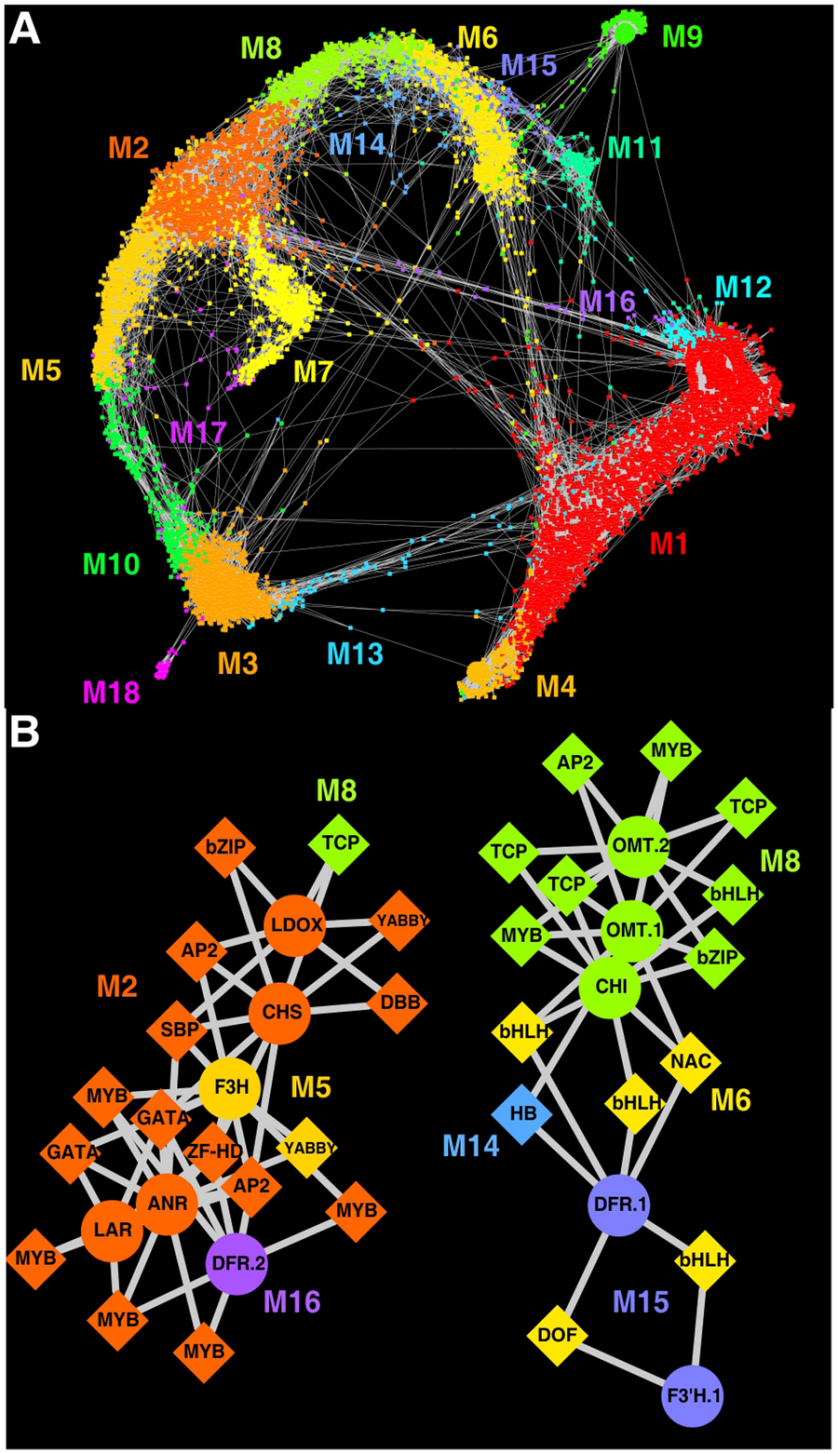
*A*. *amnicola* transcriptome co-expression networks. (**A**) Visualization of the 18 co-expression modules among the differentially expressed genes detected by WGCNA. Each node represents an individual differentially expressed gene (10,440) and each edge (577,405) represents a co-expression measure between two connected nodes, with shorter edges indicating higher co-expression. Nodes are colored by co-expression module membership and labeled M1 through M18 (Supplementary Fig. S2). (**B**) Transcription factors connected to at least two flavonoid biosynthesis unigenes when analyzing the top ten transcription factors with the highest co-expression values for each flavonoid unigene. Flavonoid biosynthesis unigenes (11) are shown as circles, and transcription factors (30) are shown as diamonds and labeled as their transcription factor class. Nodes are colored by their co-expression module membership. Edges connecting each transcription factor to a flavonoid unigene are weighted by topological overlap measures with shorter edges indicating stronger co-expression. Flavonoid biosynthesis unigene IDs are shown in Supplementary Table S5 and transcription factor unigene IDs are shown in Supplementary Table S7.

In an attempt to investigate regulatory networks that might be involved with the flavonoid pathway as well as other metabolic networks or unigenes of interest, Weighted Gene Co-expression Network Analysis (WGCNA) was applied to the *A*. *amnicola* transcriptome dataset. WGCNA takes a system level view of transcriptomic data sets and uses unsupervised hierarchical clustering to identify sets of co-expressed genes^44^. An inherent advantage of WGCNA analysis is the identification of correlations in expression levels among all unigenes instead of analyzing unigenes as individual entities as in traditional differential gene expression analysis. WGCNA enabled grouping of the 10,440* A*. *amnicola* unigene transcripts into 18 co-expression modules (Fig. 7A). Each unigene is represented by a node and is connected to other nodes by a subset of edges weighted by the highest co-expression measures (topological overlap measure, TOM) as calculated by WGCNA (577,405 connections out of nearly 55 million). Nodes are colored by co-expression module membership. These modules ranged in size from 4,066 members (module 1) to 32 members (module 18). Module eigengenes summarize the expression profile of each module (Supplementary Fig. S2). Organ specific expression modules include spadix expression in modules 3 and 18; spathe expression in modules 6, 9, and 11; leaf expression in modules 2 and 7; and root expression in modules 1, 4, and 12.

In general, unigenes with peak expression in root tissue were in the lower-right of the network (modules 1, 4, and 12 in Fig. 7A; Supplementary Fig. S2). Unigenes with peak expression in the aerial tissues were found in the arc from the lower-left corner to the top-center of the network, with spadix unigenes found in the bottom of the arc (modules 3 and 18), leaf unigenes found in the center of the arc (modules 2, 5, and 7), and spathe unigenes found in the top of the arc (modules 6, 9, 11, and 15) (Fig. 7A).

Modules found at the intersections of these regions contained unigenes expressed highly in multiple organs. For example, module 13 included unigenes with higher expression levels in roots and spadices (Fig. 7A). Similarly, module 10 lies at the intersection of spadix and leaf, module 8 lies at the intersection of leaf and spathe, module 16 spans unigenes expressed in roots to unigenes expressed in leaves, and module 11 spans both root and spathe (Fig. 7A). Thus, unigenes in modules 10, 8, 16, and 11 were co-expressed in the spadix/leaf, leaf/spathe, root/leaf, and root/spathe, respectively (Supplementary Fig. S2). WGCNA analyses not only allowed the identification of unigenes with dual-organ expression patterns as described above, but also identified clearly sample-specific patterns as shown in module 11 (Spathe-1), module 18 (Spadix-1), and module 12 (Root-3) that are likely due to differences noted between replicate samples (Supplementary Table S1, Methods).

### Identification of potential regulators of flavonoid biosynthesis genes

WGCNA co-expression network data was mined in order to identify transcription factors that were co-expressed with multiple flavonoid biosynthesis genes that might be linked to their regulation. To accomplish this, 558 putative transcription factors were identified in the *A*. *amnicola* transcriptome based on their PFAM^45^ annotations and the transcription factor family assignment rules used in the Plant Transcription Factor Database (version 3)^46^. Second, the 10 transcription factors having the highest topological overlap measure values for each identified *A*. *amnicola* flavonoid biosynthesis unigene (Supplementary Table S7) were selected. Third, the transcription factor lists for each unigene were compared, and transcription factors found in the top 10 list of two or more flavonoid biosynthesis unigenes were retained for further analysis (Supplementary Table S7). Figure 7B highlights the transcription factors showing co-expression with two or more *A*. *amnicola* flavonoid biosynthesis unigenes. Two different subnetworks were detected: *subnetwork one* centered around the flavonoid biosynthesis unigenes *CHS*, *F3H*, *DFR*.*2*, *LDOX*, *LAR*, and *ANR* and *subnetwork two* centered around *CHI*, *F3*′*H*.*1*, *DFR*.*1*, *OMT*.*1*, and *OMT*.*2*. Each flavonoid biosynthesis unigene, shown as circles, and transcription factor, shown as diamonds, are colored based on their co-expression module membership (Fig. 7A and B, Supplementary Fig. S2). The majority of the unigenes in subnetwork one (left) were members of co-expression module 2 (dark orange), representing unigenes moderately expressed in the spathe and highly expressed in the leaf, while *F3H* and a *YABBY* transcription factor gene were members of module 5 (light orange; expressed highly in leaf), *DFR*.*2* was a member of module 16 (violet; expressed highly in root and leaf), and a *TCP* transcription factor was a member of module 8 (light green; expressed highly in leaf and spathe). Meanwhile, a majority of the unigenes in subnetwork two (right) were members of module 8 (light green; expressed highly in leaf and spathe), with *F3*′*H*.*1* and *DFR*.*1* in module 15 (light violet; expressed highly in spathe), a unigene for a HB transcription factor in module 14 (light blue; high expression in spathe), and five additional transcription factors in module 6 (yellow; expressed in spathe). The overall expression pattern of subnetwork one can be summarized as having highest expression in leaf tissues with additional expression in the spathe (modules 2 and 8), spadix (module 5), and one root sample (Root-3; module 16). The expression pattern of subnetwork two consists of even expression in spathe and leaves (module 8), expression in spathe (modules 6 and 15), and expression in all organs except roots (module 14). In addition, four bHLH and seven MYB transcription factors were included in this list of flavonoid associated transcription factors. The identified *A*. *amnicola* MYB unigene (c108222_g4), with highest expression in *A*. *amnicola* leaf, is co-expressed with *F3H* and *DFR*.*2* and is homologous in sequence to the previously identified *Anthurium andraeanum* MYB gene *AaMYB1*. While the function of the identified *A*. *amnicola* MYB gene is unknown, the *A*. *andraeanum AaMYB1* homolog was able to induce anthocyanin biosynthesis alone or in combination with a maize bHLH factor in white orchid petals^47^. The co-expression of the MYB unigene transcription factor with *DFR* is also relevant since *DFR* mRNA levels have been reported to vary significantly at least in the spathe, during floral development, suggesting a regulatory role in anthocyanin biosynthesis in *A*. *andraeanum* cultivars^11, 20, 22^. A second recently described *A*. *andraeanum* MYB gene, *AaMYB2* was shown to be expressed highly in spathes and involved in anthocyanin biosynthesis by its ability to induce anthocyanin production in tobacco^19^. *AaMYB2* is similar in sequence to a unigene in this study, c108519_g1, whose expression, interestingly is characterized by highest expression in the spadix (module 3) in addition to elevated expression in the spathe, although it does not show strong co-expression with anthocyanin biosynthesis genes. In other *Anthurium* studies, *F3*′*H* was suggested to be a key gene in determining shade differences among pink and red cultivars^11, 22^. A unigene identified as *F3*′*H* (*F3'H.1*; c110285_g5) is also expressed highly in *A. amnicola* spathe tissue along with the unigene *DFR.1* (c104266_g10) and through our analyses is associated with at least one transcription factor, bHLH (c105566_g4) (Supplementary Tables S4, S5 and S7). While this co-expression data alone is not sufficient to assign definitive roles, members of these transcription factor classes have been shown to be involved in the regulation of flavonoid biosynthesis genes in other plants^48–50^ and could be linked to the same processes in *A*. *amnicola*.

## Discussion

The lack of genomic resources for the horticulturally important aroid genus *Anthurium* and for aroids in general has been an impediment towards understanding the molecular origins of commercially valuable traits and the molecular basis and evolution of aroid-specific organ form and function. In this work, the first transcriptome and gene expression data sets representing four major organs (the spathe, spadix, leaf, and root) from a single aroid and the first transcriptome dataset from a wild-type *Anthurium* species found in the pedigree of numerous commercial hybrids was assembled.

Comparison of the transcriptomes from four diverse *Anthurium amnicola* organs revealed notable differences in expression patterns of genes related to major physiological processes. Unigenes involved in photosynthesis, mostly of chloroplastic origin, were expressed more highly in leaf samples compared to the other organs, whereas expression of unigenes involved in two core metabolic pathways, glycolysis and TCA cycle were highest in the spadix. Unigenes involved in interactions with microbes were highly expressed in the root. Unigenes involved in pigment biosynthesis, including unigenes assigned to the phenylpropanoid and flavonoid biosynthetic pathways, were also expressed in organ specific patterns. For example, unigenes assigned to the anthocyanin pathway were expressed at the highest levels in the leaves and spathes while unigenes for steps in the carotenoid biosynthetic pathway associated with the synthesis of abscisic acid were highest in the spadix. At least two unigenes associated with chlorophyll catabolism were highly expressed in both the spadix and spathe, suggesting an active regulation of green color in these organs. Additionally, our RNA sequence expression data identified a number of potential organ-specific unigenes. These included transcription factors and homeotic genes that may comprise some of the first molecular clues to aroid organ, organ-specific expression, formation and identity. The basis for the unique organ-specific unigene expression patterns and potential organ-specific unigenes, particularly in the aroid-specific spadix and spathe organs, is unknown at this time given the lack of comparable model systems. However, the expression patterns of these unigenes allow the design of testable hypotheses towards answering questions on gene function in these aroid-specific organs.

Among four previous transcriptome studies on *Anthurium*, all performed on ornamental cultivars, one examined cold stress of whole seedlings^14^, while three examined the molecular basis for color in *Anthurium*. Of the three focused on plant color, the first examined expression differences between leaves and spathes of leaf color mutants^18^, the second, expression differences between spathes of a red-spathed cultivar and its anthocyanin-loss mutant^16^, while the third utilized a mixed floral (spathe and spadix) and leaf transcriptome to identify a anthocyanin related MYB transcription factor^19^. Roots and spadices were not specifically examined in any of the previous studies. In the study examining gene expression in the leaves and spathes of leaf color mutants, the specific causative mutation was not identified, although *DFR* and *F3H* were identified as key genes for anthocyanin production in the cultivar studied. In the study examining gene expression differences between the pigmented and anthocyanin-loss mutant spathes, upregulation of *AN2*, a regulatory element belonging to the MYB transcription factor family that was shown to repress anthocyanin biosynthesis when expressed heterologously in Arabidopsis, and downregulation of the gene *UFGT* encoding UDP-glucose: flavonoid 3-O-glucosyltransferase which is involved in flavonoid glycosylation, was observed in the *Anthurium* mutant lacking anthocyanin compared to the wild type cultivar. In the third study, a novel Anthurium MYB transcription factor gene, *AaMYB2* was identified and shown to be highly expressed in the spathe as well as able to induce anthocyanin accumulation in tobacco.

The use of WGCNA to identify co-expression patterns among the four different tissues was especially key for identifying potential regulatory elements for pathways of interest. This analysis enabled the identification of a MYB transcription factor gene homologous to *A*. *andraeanum AaMYB1* that is expressed at relatively low levels in all organs, but most highly in *A*. *amnicola* leaves, thereby identifying it as a gene co-expressed with flavonoid pathway genes *DFR*.*2* and *F3H*. Similarly, a gene for a bHLH transcription factor was found to be co-expressed with potential target anthocyanin biosynthesis pathway unigenes, *F3'H.1* and *DFR.1*, expressed highly in spathes. The identification of candidate transcription factors whose expression patterns correlate with flavonoid biosynthesis genes contributes new information to previous genetic and molecular models on the regulation of anthocyanin biosynthesis in various *Anthurium* cultivars^8–11, 16, 18, 20, 22^. A unigene, c108519_g1, similar in amino acid sequence to a gene *AaMYB2*, previously implicated in anthocyanin biosynthesis in spathes in an *A*. *andraeanum* cultivar was identified in the *A*. *amnicola* transcriptome, but unlike *AaMYB2* was also highly expressed in the *A*. *amnicola* spadix consistent with pigment phenotypes of both organs in *A*. *amnicola* and a role in anthocyanin biosynthesis in these tissues. However, this transcript was not highly correlated with the expression of anthocyanin biosynthesis genes. Failure to identify an *A*. *amnicola AaMYB2*-like gene by coexpression analyses may indicate that coordinated expression of transcription factor and target genes do not fully explain regulation of this biosynthetic pathway, or that regulation differs by organ, tissue, cultivar or species.

In this study, we show that flavonoid and anthocyanin genes were differentially expressed in all four organs of *A*. *amnicola* with various copies of *DFR* and *F3*′*H* genes expressed differentially across the four organs. For example, *DFR*.*2* appears to be highly expressed in leaves and roots whereas *DFR*.*1* is highly expressed in the spathe and its expression level was not detectable in roots. Of the three *F3*′*H* unigenes, *F3*′*H*.*1* is expressed highest in the spathe, *F3*′*H*.*2* is expressed highest in the root, and *F3*′*H*.*3* is highest in the spadix. Thus, this work has clearly revealed the existence of overlapping as well as distinct gene networks governing flavonoid and anthocyanin biosynthesis in different organs of an *Anthurium* species. Given our knowledge of the existence of unique, distinctly expressed *Anthurium* MYB factors each shown to induce anthocyanin accumulation in heterologous systems, information on gene copy number and gene networks should be useful for further understanding of pigment regulation at the whole plant level in *Anthurium* species, ornamental cultivars and hybrids.

Altogether, our current transcriptome analyses will serve as a foundation for future research targeted at establishing metabolic networks and genes that impact important traits in *Anthurium* as well as provide underpinnings for understanding the differences in physiology, function and formation of different organs still poorly understood at the molecular level in Aroids.

## Methods

### Sample collection from *Anthurium amnicola*

Samples for RNA isolation were collected from mature flowering vegetative clones of *A*. *amnicola*, University of Hawaii at Manoa (UHM) accession A667. The *A*. *amnicola* were grown in a shadehouse at the UHM, Waiakea Experiment Station (Lat: 19.64424, Lon:-155.07957, elevation, 183.23 meters) located in Hilo, Hawaii, annual precipitation approximately 322 cm, mean annual air temperature 21–23 °C. Plants were grown in 15.24 cm round pots in black cinder medium on a raised bench under natural lighting with 80% saran shade, watered daily with overhead irrigation in addition to natural precipitation and fertilized with 13-13-13 slow release fertilizer every four months. A667 was acquired as an *Anthurium amnicola* plant originating from seed-propagated material from Christchurch Botanic Gardens, Christchurch, New Zealand^51^. Leaves, spathes, spadices and roots were rapidly harvested, dissected with razor blades to isolate organ tissue, surface cleaned with dH_2_O and Kimwipes and quickly frozen directly in liquid nitrogen on site in the shadehouse. Spathe and spadix samples were harvested from the same floral stem and represent a narrow developmental time period spanning a “late unfurling” state (between stage 4 and 5 defined for *A*. *andraeanum* ^20^; samples Spathe-1 and Spadix-1, Supplementary Table S1) and a “recurved” state, defined here as a stage when the spathe is fully unfurled evidenced by recurving at the tip, but prior to protrusion of stigma and stamen (Fig. 1B; samples Spathe-3 and Spadix-3, Supplementary Table S1). Leaf samples used for RNA isolation were newly expanded and supple (Supplementary Table S1). Roots from below the pot line in cinder were harvested, rinsed in a large volume of water to remove excess debris and wiped dry with Kimwipes prior to freezing in liquid nitrogen. Harvest of the entire organ sample set was completed between 10:45–11:30 a.m. on the same day, August 19, 2014. Dissected, frozen organ samples were stored at −80 °C. Total RNA was isolated from tissue (~250 mg fresh weight) collected from whole spathes, spadices, leaves, and roots using the Purelink RNA mini Kit (Ambion by Life Technologies) with minor modifications. The samples were DNase treated and RNA integrity was assessed by running samples on a Bioanalyzer.

### RNA extraction and high-throughput sequencing

At least 1.0 µg of total RNA of concentrations of approximately 20 ng/µL from the purified stocks were prepared for sequencing using the TruSeq Stranded mRNA Library Preparation Kit (Illumina Inc., San Diego, CA, USA). The library pool was sequenced on an Illumina HiSeq 2500 platform to a depth of approximately 20 million 151 bp paired-end reads per sample. All raw reads were submitted to the NCBI Sequence Read Archive under accession numbers SRS979605-SRS979628 described in Biosamples SAMN03839010-SAMN03839021 associated with BioProject PRJNA288827 (see Supplementary Table S1 for each sample’s accessions).

### *In silico* library normalization and *de novo* transcriptome assembly

Raw reads were filtered using Trimmomatic (v. 0.32) using the options “ILLUMINACLIP:TruSeq3-PE.fa:2:30:10 LEADING:5 TRAILING:5 SLIDINGWINDOW:4:10 MINLEN:36”. These quality-filtered reads were then normalized to reduce redundant read data and discard read errors using Trinity's *in silico* normalization tool, with a kmer size of 25 and maximum read coverage of 40. The resulting normalized reads were used in Trinity's *de novo* transcriptome assembly pipeline (r20140717)^52^. The Trinity pipeline (Inchworm, Chrysalis, and Butterfly) was executed using default parameters, implementing the “–SS_lib_type RF” flag to calculate the assembly based on strand-specific sequencing libraries and using a minimum kmer coverage of 1^53^.

### Assembly filtering, open reading frame prediction, and functional annotation

After graph reconstruction with Butterfly, assembled transcripts were grouped into unigene components, which represent putative unigenes, with each transcript representing putative isoforms across the unigene model for which it was assigned. While many full-length transcripts were expected to be present, it is likely that the assembly also consisted of erroneous contigs, partial transcript fragments, poorly supported isoforms, and non-coding RNA molecules. This collection of sequences was filtered to retain only contigs containing full-length or near full-length transcripts and coding regions. Furthermore, only isoforms that were represented at a minimum expression level based on read abundance were retained. To accomplish this, several steps were taken.

First, pooled unnormalized reads were aligned to the unfiltered Trinity.fasta transcript file using bowtie2 (v. 2.0.0) with the ‘align_and_estimate_abundance.pl’ script distributed with Trinity. The abundance of each transcript was calculated using RSEM (RNA-Seq by Expectation Maximization) v1.2.0 on a per transcript isoform and per unigene basis^54^ and the percent composition of each transcript component of each gene was calculated. Next, coding regions were predicted using Transdecoder (v 2.0) with default parameters with the addition of HMMER (v 3.0) to identify Pfam-A domains, which were used to provide further evidence to support coding region predictions^55^. The transcriptome was filtered to contain only transcripts that contained a predicted open reading frame and transcripts with a TPM (Transcripts per Million) abundance greater than 0.5.

To aid in functional assignments, all predicted proteins were subjected to analysis with InterProScan5 searching all available databases including gene ontology and InterPro term lookup. In addition, predicted proteins were subjected to BLASTp searches against the UniProtKB/SwissProt database (downloaded 10 November 2013). Transcripts were also further analyzed using the Trinotate functional annotation pipeline (Trinotate_r20140708) (Supplementary Table S8) and protein coding transcripts were assigned to metabolic pathways and KEGG^28^ orthology terms using KAAS (KEGG automatic annotation server)^56^. The amino acid sequences were assigned to pathways using the bi-directional best hit method for partial genomes and the *Oryza sativa* genome as a reference^57^ (Supplementary Table S3A).

Annie (http://genomeannotation.github.io/) was used to generate consensus annotations, which assigns gene names and products using top SwissProt blast matches and performs database cross-referencing from InterProScan5 results. The resulting annotation file, RSEM read abundance matrix, and GFF3 (General Feature Format, version 3) annotation file from Transdecoder, were used as input to Transvestigator to generate annotations in.gff3 and.tbl format (described at http://genomeannotation.github.io/). The fully annotated transcriptome assembly was submitted to NCBI under Transcriptome Shotgun Assembly (TSA) GDJX00000000 associated with Bioproject PRJNA288827.

### Read library mapping and differential expression analysis

Because the Trinity assembler is able to accurately predict splice isoforms, unigene and isoform level expression quantification was performed using the RSEM software package. The filtered transcript set (TSA GDJX00000000) was used as the reference in this analysis, which only contains transcripts containing likely coding sequence (full and partial ORFs) to avoid skewing results with non-coding and fragmented data. Quality filtered reads were mapped to the reference transcriptome assembly using bowtie2 (v 2.0.0)^58^ with the ‘align_and_estimate_abundance.pl’ script. Expected read counts were calculated using RSEM. Prior to heatmap and network analyses, read counts for each gene were normalized using the trimmed mean of M values method (TMM) and fragments per kilobase per million mapped reads (FPKM) (Supplementary Tables S9A and B). The TMM values were subjected to hierarchical clustering analysis based on Euclidean distances using the complete agglomeration algorithms. FPKM values were used to summarize expression patterns of each cluster.

### qPCR validation

cDNA was prepared from 25 ng total RNA using the SuperScript III First-Strand Synthesis System for RT-PCR kit (Invitrogen™, ThermoFisher Scientific) and random hexamers per manufacturer’s instructions. qPCR was performed on each sample in triplicate using the same 12 samples that were used to construct the RNA-Seq libraries. Each reaction included template cDNA (corresponding to equivalent of 0.5 ng starting total RNA per reaction), target gene primers (Supplementary Table S6), and the SYBR Select Master Mix (Applied Biosystems™, ThermoFisher Scientific) and the analysis was performed in an Applied Biosystems QuantStudio 5 Real-Time PCR system following the manufacturer’s instructions.

### Co-expression network analysis

Co-expression networks were generated using the R^59^ package Weighted Gene Co-expression Network Analysis (WGCNA, version 1.43)^44, 60^. TMM normalized FPKM counts were used to identify gene sets showing similar expression patterns across the various *Anthurium* tissue samples. In order to meet the requisite scale-free topology criteria for WGCNA, a soft threshold (β) value of 20 was used in the adjacency calculation. The following parameters were used in the creation of the signed co-expression modules: maxBlockSize = 11000, midModuleSize = 30, deepSplit = 2, and mergeCutHeight = 0.2. The expression patterns of the resulting co-expression modules are each summarized by a module eigengene, which is analogous to the first principal component of a given module and its expression profile across all tissues sampled.

### Network visualization

Topological overlap measures (TOM) calculated by WGCNA were used as co-expression measures for gene pairs, with a higher value indication higher co-expression. The total network size (10,400 × 10,400 matrix) was reduced in size by retaining the four highest TOM values for every gene as well as the top 1% overall TOM values. This resulted in a final network of 10,400 nodes (differentially expressed genes) connected by 577,405 edges (TOM values). Cytoscape (version 3.2.0)^61^ was used to visualize the resulting network using the AllegroLayout plugin with an edge-weighted Allegro Fruchterman-Reingold layout algorithm.

## Electronic supplementary material

Supplementary Information Summary

Supplementary Figure S1

Supplementary Figure S2

Supplementary Tables
