# Supplementary material for: Organ-specific transcriptome profiling of metabolic and pigment biosynthesis pathways in the floral ornamental progenitor species Anthurium amnicola Dressler
Source: Sci Rep. 2017 May 4;7:1596. doi: 10.1038/s41598-017-00808-2 (PMC5431427; doi:10.1038/s41598-017-00808-2)
Supplement: Supplementary file 1 — Supplementary Information Summary [file 41598_2017_808_MOESM1_ESM.pdf]

Organ-specific transcriptome profiling of metabolic and pigment biosynthesis pathways in the floral ornamental progenitor species *Anthurium amnicola* Dressler

Jon Y. Suzuki[1*], Teresita D. Amore[2], Bernarda Calla[1δ], Nathan A. Palmer[3], Erin D. Scully[4], Scott E. Sattler[3,5], Gautam Sarath[3,5], Joanne S. Lichty[2], Roxana Y. Myers[1], Lisa M. Keith[1], Tracie K. Matsumoto[1], Scott M. Geib[1]

Supplementary Information Summary

Supplementary Table S1 - *A. amnicola* sample and raw data statistics and database accessions.

Supplementary Table S2 - *A. amnicola* transcriptome assembly and annotation statistics.

Supplementary Table S3A - KEGG metabolic pathways assignment using the bi-directional best hit method for partial genomes and the *Oryza sativa* genome as a reference. KEGG pathway assignments for protein coding transcripts assembled from *Anthurium.*

Supplementary Table S3B - Unique KEGG ontology terms identified in the *A. amnicola* transcriptome and select plant genomes.

Supplementary Table S4 - Differential expression results. List of 10,440 genes showing differential expression across pairwise comparisons.

Supplementary Table S5 - Flavonoid biosynthesis genes identified in *Anthurium amnicola* transcriptome assembly.

Supplementary Table S6 - qPCR genes and primers.

Supplementary Table S7 - Transcription factors sharing co-expression with multiple *Anthurium amnicola* flavonoid biosynthesis genes.

Supplementary Table S8 - Annotation table of all transcripts. Trinotate derived annotations. (accessible at: https://figshare.com/articles/Annotation_Table_for_Transcriptome_Assembly/3493505, Annotation Table for Transcriptome Assembly 20.07.2016, 13:32 (GMT) by Jon Y. Suzuki, Teresita D. Amore, Bernarda Calla, Nathan A. Palmer, Erin D. Scully, Scott E. Sattler, Gautam Sarath, Joanne S. Lichty, Roxana Y. Myers, Lisa M. Keith, Tracie K. Matsumoto, Scott Geib).

Supplementary Table S9A - Raw read counts matrix.

Supplementary Table S9B - TMM normalized FPKM matrix. Normalized counts used for differential expression analyses.

Supplementary Figure S1 - qPCR validation of select target genes. qPCR ΔCt values and RNA-seq data correlation for seven *A. amnicola* metabolic pathway unigenes corresponding to genes *ACLY*, *DLAT*, *ALDO*, *HEMD*, *HST*, *NCED*, *CHI* (Supplementary Table S6).

Supplementary Figure S2 - Network co-expression module profiles. Organ specific expression profiles for the 18 co-expression modules in Fig. 7.
