# Supplementary material for: Organ-specific transcriptome profiling of metabolic and pigment biosynthesis pathways in the floral ornamental progenitor species Anthurium amnicola Dressler
Source: Sci Rep. 2017 May 4;7:1596. doi: 10.1038/s41598-017-00808-2 (PMC5431427; doi:10.1038/s41598-017-00808-2)
Supplement: Supplementary file 2 — Supplementary Figure S1 [file 41598_2017_808_MOESM2_ESM.pdf]

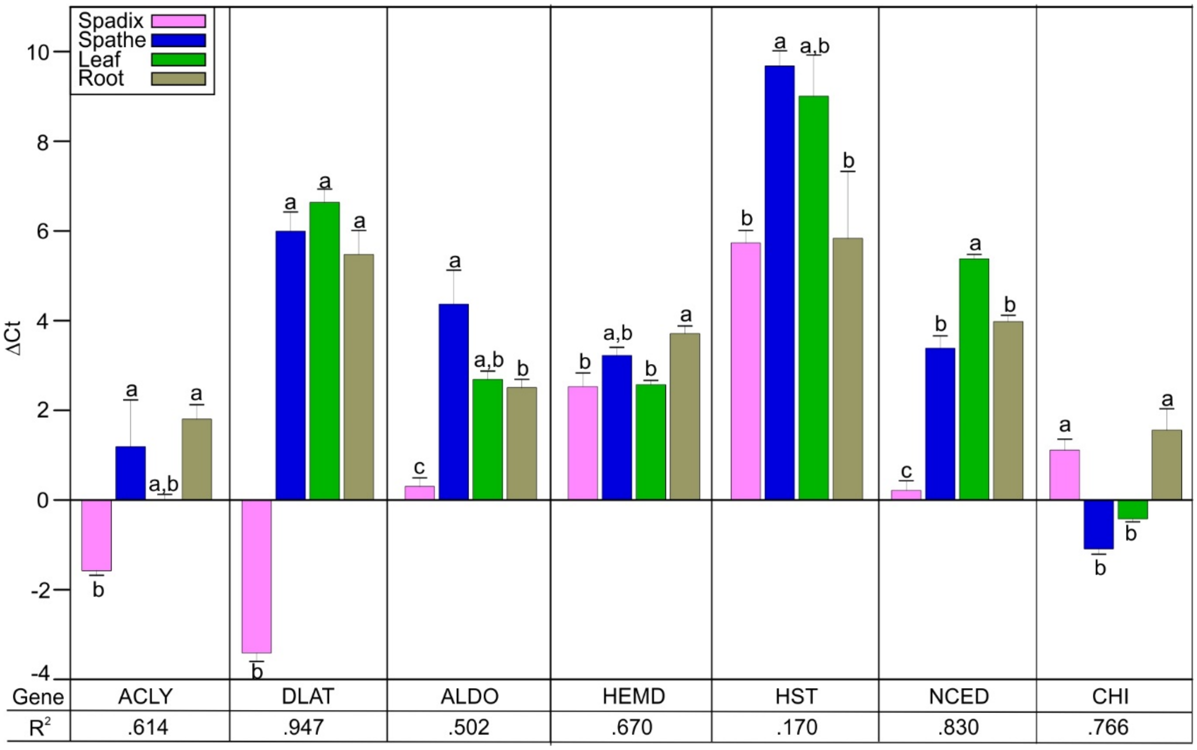

Supplementary Figure S1 –qPCR validation of select target genes. qPCR ΔCt values for seven *A. amnicola* metabolic pathway unigenes corresponding to genes *ACLY*, *DLAT*, *ALDO*, *HEMD*, *HST*, *NCED*, *CHI* (Supplementary Table S6) based on the mean of three samples each of four tissue types, spadix (pink), spathe (blue), leaf (green) and root (brown) are shown. ΔCt values were calculated using two reference genes *ACT* and *IF3B*. RNA-seq data correlation, represented by R^2 was calculated by comparing qPCR ΔCt and RNA-Seq TMM normalized FPKM values for each gene across all 12 (three samples for each of the four tissue types) samples. Error bars denote standard error; different letter designations denote significant ΔCt value differences.
