# Supplementary material for: Organ-specific transcriptome profiling of metabolic and pigment biosynthesis pathways in the floral ornamental progenitor species Anthurium amnicola Dressler
Source: Sci Rep. 2017 May 4;7:1596. doi: 10.1038/s41598-017-00808-2 (PMC5431427; doi:10.1038/s41598-017-00808-2)
Supplement: Supplementary file 3 — Supplementary Figure S2 [file 41598_2017_808_MOESM3_ESM.pdf]

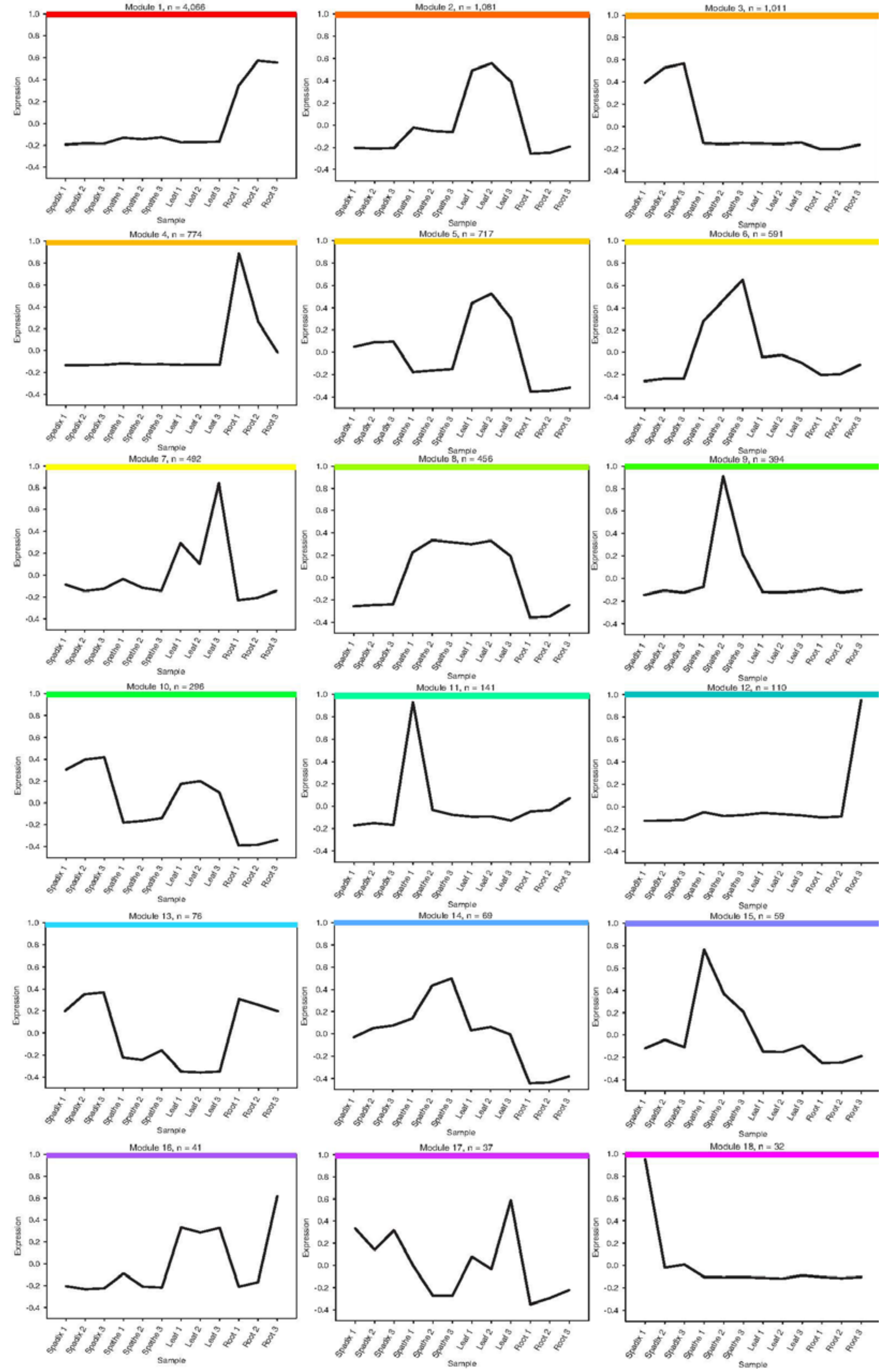

Supplementary Figure S2 – Network co-expression module profiles. Organ specific expression profiles for the 18 co-expression modules in Fig. 7. The colored bar above each plot corresponds to the module color found in Fig. 7 and the number of genes (n) are included at the top of each plot.
